# Metastatic Small Cell Carcinoma of the Urinary Bladder That Recurred in the Vagina 6 Years after Radical Cystectomy: A Case Report

**DOI:** 10.1155/2018/3069294

**Published:** 2018-10-24

**Authors:** Makoto Isono, Keiichi Ito, Shinsuke Hamada, Masahiro Takahashi, Hidenori Sasa, Hideyuki Shimazaki, Tomohiko Asano

**Affiliations:** ^1^Department of Urology, National Defense Medical College, 3-2 Namiki, Tokorozawa, Saitama 359-8513, Japan; ^2^Department of Obstetrics and Gynecology, National Defense Medical College, 3-2 Namiki, Tokorozawa, Saitama 359-8513, Japan; ^3^Department of Laboratory Medicine, National Defense Medical College, 3-2 Namiki, Tokorozawa, Saitama 359-8513, Japan

## Abstract

Small cell carcinoma (SCC) of the urinary bladder is highly aggressive and portends a poor outcome. Herein, we report a patient with recurrent SCC of the urinary bladder who experienced an unusually long-term disease-free duration after radical cystectomy. The patient was a 60-year-old woman who had undergone transurethral resection followed by radical cystectomy for muscle-invasive bladder cancer (high-grade urothelial carcinoma with adenocarcinomatous differentiation) 6 years prior; the surgical specimen had a negative surgical margin. She was referred to our hospital because of continuous bleeding from her vagina. Magnetic resonance imaging showed a mass located at the anterior wall of her residual vagina, a biopsy of which confirmed a pathological diagnosis of adenocarcinoma. The vaginal tumor and a section of the sigmoid colon were resected* en bloc* and were pathologically diagnosed as adenocarcinoma and SCC. We reevaluated the initial transurethral resection specimen and found SCC with foci of adenocarcinoma concomitant with high-grade urothelial carcinoma. Local recurrence and metastasis at the pelvic bone occurred 4 months later; although radiation therapy was performed, she died of the progressive disease.

## 1. Introduction

Small cell carcinoma (SCC) of the urinary bladder is a rare disease, accounting for less than 1% of all primary bladder tumors [1]. The diagnosis of SCC is based on criteria established by the World Health Organization classification system [2] and can be facilitated by immunohistochemical staining. SCC is a poorly differentiated tumor, usually progresses rapidly, and carries a poorer prognosis than urothelial carcinoma (UC) of the urinary bladder. According to a recent investigation, the median overall survival in patients with SCC of the bladder who were metastasis-free at primary diagnosis was 8.3 months [3]. Because of the disease's rarity, no standard management has yet been proposed for this disease.

We report an unusual episode of a patient with SCC of the urinary bladder in whom the tumor recurred in the residual vagina after a relatively lengthy disease-free duration following radical cystectomy that was performed with a curative surgical margin.

## 2. Case Presentation

**Figure 1:**
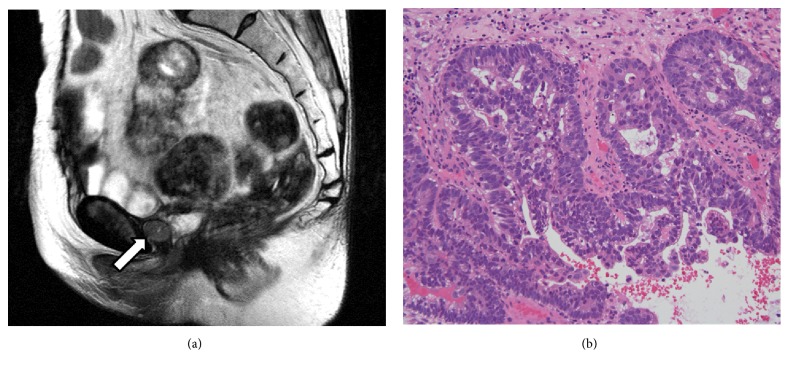
(a) MRI imaging showed a tumor at the anterior wall of the residual vagina (arrow). (b) Microscopic examination of biopsy for the vaginal tumor showed adenocarcinoma (hematoxylin-eosin stain, original magnification: ×400).

**Figure 2:**
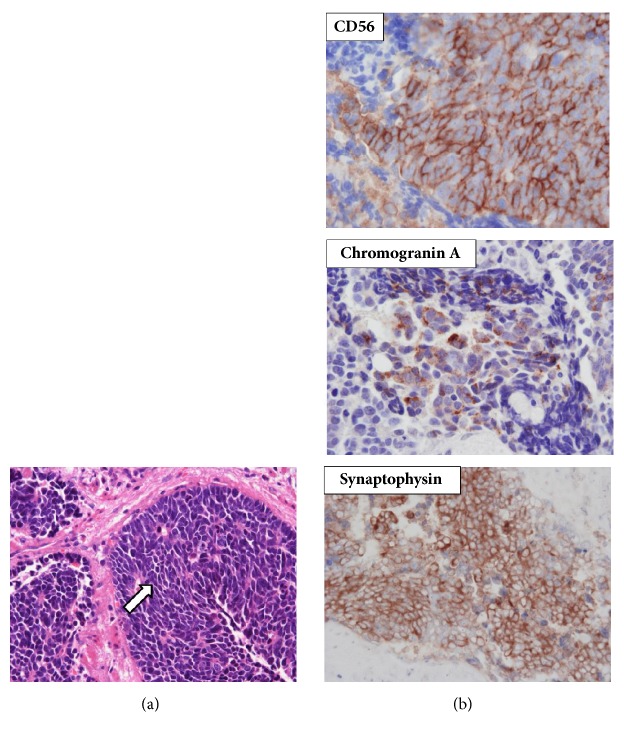
Microscopic finding of the vaginal tumor. (a) There were round to spindle-shaped cells having scanty cytoplasm and high mitotic activity (arrow). The pathological diagnosis was small cell carcinoma (hematoxylin-eosin stain, original magnification: ×400). (b) On immunohistochemistry, the tumor was found to be positive for CD56, chromogranin A and synaptophysin (original magnifications: ×400). Positivity for these makers suggested that the tumor was small cell carcinoma.

**Figure 3:**
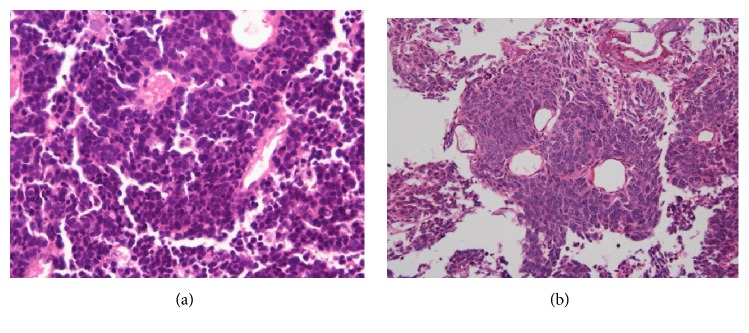
Microscopic finding of the initial TURBT. (a) We examined the TURBT specimen again and found that there were SCC and foci of adenocaricinoma in the superficial layer of the specimen (hematoxylin-eosin stain, original magnification: ×400). This result indicated that the vaginal tumor was a recurrence of SCC and adenocarcinoma of the urinary bladder. (b) High-grade UC in the TURBT specimen (hematoxylin-eosin stain, original magnification: ×100).

A 60-year-old woman underwent transurethral resection of bladder tumor (TURBT) at our institute in 2004; her pathological diagnosis was a high-grade UC with adenocarcinomatous differentiation (pT2a, G2>G3). Radical cystectomy was conducted. Only carcinoma in situ (CIS) was found in the surgical specimen, and the surgical margin was negative. There was no cancer cell infiltration in the resected uterus or anterior wall of the vagina, and no lymph node involvement was detected. The patient developed continuous pain and bleeding from the residual vagina in 2010, and a tumor was found in the residual vagina; magnetic resonance imaging (MRI) showed it to be located on the anterior wall (Figure 1(a)). A biopsy of the tumor revealed a pathological diagnosis of adenocarcinoma (Figure 1(b)). Computed tomography (CT) and bone scintigraphy revealed no metastasis. Based on a preoperative diagnosis of a primary adenocarcinoma occurring on the residual vagina, tumor resection was performed (Figure 2(a)). The sigmoid colon was partially resected as it was strongly adherent to the tumor. On pathological examination, adenocarcinoma and SCC were detected (Figure 2(b)); on immunohistochemistry, sections of the tumor were positive for the SCC markers CD56, chromogranin A, and synaptophysin and were negative for the urothelial carcinoma markers GATA-3, p63, uroplakin, thrombomodulin, and 34*β*E12. We then reexamined the original TURBT specimen and confirmed the presence of SCC (Figure 3). Adenocarcinoma and SCC were mostly present in the superficial layer of the TURBT specimen, while high-grade UC was found in the deeper layers where muscle invasion was present. Based on these findings, the tumor was diagnosed as a recurring bladder tumor. Local recurrence and pelvic bone metastasis were detected via MRI 3 months after the patient underwent surgical resection of the vaginal recurrence, whereupon she underwent radiation therapy (52 Gy, 26 fractions). She developed ileus in January 2011 and underwent release surgery. Subsequently, multiple lung metastases and local recurrence in the pelvis developed in June, and she died of disease progression the following month.

## 3. Discussion

Primary SCC of the urinary bladder is a rare bladder malignancy with an incidence rate of less than 1% [1]. It has a strong male predominance, with a male:female ratio of 3–5.1:1 [1, 4]. The pathogenesis of primary SCC of the urinary bladder is not well defined and is postulated to involve carcinogenesis in multipotential epithelial reserve cells, as it often coexists with UC and/or adenocarcinoma [1]. Abrahams et al. reported that 70% of SCCs coexist with UC, while only 8% and 10% of all SCC coexist with adenocarcinoma and squamous carcinoma cases, respectively [5].

There is little consensus on optimal treatment guidelines for urinary bladder SCC because of its rarity. Various combinations of surgery, radiotherapy, and chemotherapy have been used [6]. The most common chemotherapy regimens used are platinum-based regimens similar to those administered to patients with SCC of the lung [7]. Radical cystectomy is widely performed in patients with no evidence of metastasis [1]. However, Cheng et al. questioned the efficacy of radical cystectomy because of no difference in survival outcomes between patients who underwent surgery and those who did not [8]. According to a previously published Surveillance, Epidemiology and End Results (SEER)-based analysis, surgical treatment alone without multimodal therapy (chemotherapy and/or radiation therapy) is not adequate for SCC of the bladder [9]. In our patient, adjuvant chemotherapy was not administered because only CIS was found in the radical cystectomy specimen, even though high-grade UC and adenocarcinomatous differentiation were present in the TURBT specimen.

The outcome for patients with SCC of the urinary bladder is extremely poor. Patients with SCC tend to progress quickly and develop metastases [10], and their prognosis is poor even with limited-stage disease [8]. Survival rates are also low; for example, Patel et al. reported a 3-year overall survival rate of 33% in their patients [11]. Even with aggressive multimodal treatments, Choong et al. reported 5-year survival rates for patients with stage II, III, and IV disease of 63.6%, 15.4%, and 10.5%, respectively [1]. In certain series, patients with pure SCC of the bladder tended to have poorer outcomes than those with SCC and UC combined [12, 13]. Meanwhile, Kaushik et al. did not identify any statistical differences in overall or cancer-specific survival rates between patients with mixed histology and those with pure SCC [14]. In our patient, SCC, adenocarcinoma, and high-grade UC coexisted in the TURBT specimen, and only UC (CIS) remained in the radical cystectomy specimen. SCC and adenocarcinoma were located in the superficial layer of the specimen, while high-grade UC was located in the layer of muscle invasion.

The patient had an unusually long disease-free duration after the radical cystectomy. No specific randomized control trial has assessed the risk factors for recurrence of urinary bladder SCC. Retrospective analyses of the overall survival and risk factors for recurrence of other tumors revealed that advanced stage was an independent risk factor for overall survival and disease-free survival [15, 16]. The late recurrence observed in our case, despite the biological aggressiveness of SCC, could be because the SCC component existed only at the superficial layer of the bladder and was completely resected during TURBT. It is not clear how the tumor cells remained localized as the cystectomy specimen comprised only the CIS lesion and its surgical margin was pathologically negative. Furthermore, SCC and adenocarcinoma were located in the superficial layer, while high-grade UC was located deep in the TURBT specimen. Hence, we cannot conclude whether the vaginal recurrence was a local recurrence or metastatic disease.

It is unclear whether we would have performed adjuvant chemotherapy after radical cystectomy if the presence of SCC had been diagnosed at the time of TURBT. We may have administered adjuvant chemotherapy because of the highly malignant potential of SCC even though it was located in the superficial layer. However, the pathology of the TURBT specimen was not pure SCC, and only the CIS lesion was present in radical cystectomy specimen. Additionally, the efficacy of adjuvant chemotherapy for SCC of the urinary bladder has not been confirmed. Kaushik et al. suggested that the observed improvement in overall survival in their patients who received adjuvant chemotherapy should be interpreted with caution because only patients who achieved adequate recovery after surgery and had relatively good renal function were selected for adjuvant chemotherapy [14]. Thus, the role of adjuvant chemotherapy for patients with SCC of the urinary bladder at the time of radical cystectomy requires further investigation.

## 4. Conclusion

Although SCC was present only in the superficial layer of the initial TURBT specimen and was not confirmed in the cystectomy specimen, SCC recurred in the residual vagina 6 years after the cystectomy. It is very rare because such a highly malignant tumor should recur after a relatively long disease-free duration.

## Abbreviations

(SCC): Small cell carcinoma
(UC): Urothelial carcinoma
(CIS): Carcinoma in situ
(TURBT): Transurethral resection of bladder tumor
(MRI): Magnetic resonance imaging
(CT): Computed tomography.
